# King Edward's Hospital Fund for London Statistics (First Notice)

**Published:** 1914-10-10

**Authors:** 


### HOSPITAL ECONOMICS.

### King Edward's Hospital Fund for London Statistics. (First Notice).

A decade represents a period of time adequate to test the value or otherwise of a new enterprise. The 1913 Annual Statistical Report is the eleventh of the series prepared by Mr. Maynard, the secretary of the King's Fund, from the published accounts of 106 London hospitals and from the returns made to the Fund. We published last week, page 20, a table setting forth the number and particulars of 101 of these hospitals in each of ten sections. The report deals with 9,171 hospital beds in average daily occupation in 1913. These beds accommodated 134,749 in-patients during that year, and the patients who attended the out-patient department numbered 1,329,567. These figures are valuable, but they do not include those of St. Bartholomew's Hospital. They show that just over 20 per cent., or one in five, of the population of Greater London received treatment at some London hospital during 1913. The Londoners so treated amounted probably to some one in four, because the figures include all patients, and large numbers from extra-metropolitan districts obtain treatment at the London hospitals every year.

The report of the honorary secretaries, Sir Savile Crossley, Bart., and Mr. John G. Griffiths, to the governors of the Fund, forcibly demonstrates the complete success of King Edward's Hospital Fund, and the reason why its work is gratefully recognised throughout the hospital world. The statistics issued by this Fund are based upon accurate data rendered possible by the co-operation of the managers of the hospitals. No fact or figure is published before it has been exhaustively tested, and no conclusion is put forward hastily or at all unless all the facts in regard to it are complete. Thus the report shows that during 1913 the increased cost per bed occupied and per attendance of out-patients collectively represents about £27,000. But it is insisted that "the continued increase in cost is not necessarily attributable to a disregard of that economy which, in previous years, had created very large savings." It is added that this increase in cost is not shared by all the groups of hospitals dealt with in such a report nor by all individual hospitals in the groups that collectively show an increase. Special tables are given on pages 12-13 to prevent the possibility of misapprehension on the question whether any rise anywhere is greater or less than the average increase amongst comparable hospitals generally. Another good point is the emphasis placed on the fact that the comparisons of cost are made with the average, and it is not suggested that the lowest cost is of necessity to be held up as a model. Excellent, too, is the insistence that the object of these reports is not in any way to discourage expenditure that is held to be necessary in carrying on the work, or from efforts to improve the quality of the work and its range, or as resulting from modern discoveries. The object is to assist the hospitals to discover and check, where it exists, any tendency to wasteful or unnecessary expenditure, that the maximum proportion of the funds may be directed to the maintenance or increase of efficiency. We have had the pleasure of dealing in successive years with each statistical report previously issued. In our criticisms we have tried to make them suggestive and helpful and so worthy of careful consideration. We are glad to observe that some of The Hospital's suggestions have been reduced to practice, a fact which we accept as evidence that our critic has approached the subject from the same standpoint as those responsible for the management of the King's Hospital Fund.

We have not yet had time to study fully the Eleventh Statistical Report for the year 1913, but it contains further and forcible evidence of the progressive value of the work done by the King's Fund through the admirable system adopted. It reflects credit upon Mr. Maynard, who has prepared this report, and who has abundantly justified the appointment as secretary of the Fund of a gentleman who has studied and been practically trained in all the ramifications of statistics and economic science. This training gave him a keen appreciation of the importance of treating such matters judicially. Another source of strength to the King's Hospital Fund has been its policy to avoid the possibility of coming into competition with the managers of the voluntary hospitals by making special efforts to raise income directly through the Fund. The work done, its excellence, and the increase in authority which has resulted to the managers of the Fund, have brought spontaneous contributions and gifts which have produced a steady increase in the funds placed at their disposal for distribution.

The results attained through the Fund's instrumentality since it was founded by our beloved King Edward VII. in 1897 have proved momentous for good not only to the hospitals, but to the population of London and, indeed, to the nation. To its establishment and successful work the nation feels justly that the continuous extension and success of the voluntary system of British hospitals is largely due. We are living in extraordinary times, and no members of our race ever enjoyed greater privileges or higher responsibilities. Happily, nation and Empire are united as one man in defence of freedom and the upholding of the rights of the smallest and humblest nations. It would be a worthy exhibition of the depth and soundness of the spirit which animates all classes if the present period when business is necessarily largely interfered with, should be availed of to organise in every great city and county throughout the United Kingdom a Hospital Council with the object of empowering it to carry out there a system based upon the experience and policy so successfully followed by the King's Hospital Fund.

